# Sanitary

**Published:** 1879-05

**Authors:** 


### SANITARY.

In the report of the English Parliamentary Commission, instituted for the purpose of testing the best method of utilizing human manure from sewers, the mode of carrying the sewerage through pipes and depositing it upon waste lands, for the purpose of irrigation, was considered the best.

It is by no means a favorable indication when the same generation which produced an invention has also to admit its great defects. The originators are unwilling, or, at least, slow, to admit their mistake, which is easily understood, and, in a great measure, excused, and this illustrated by the fact that after all the voluminous writings and arguments in favor of the London system the very Parliamentary Commission, instituted for its investigation, did unanimously condemn the whole system of sewerage.

Nothing was more natural than that great beneficial results were anticipated from a system which, superficially looked upon, was based upon such simple principles. "Let," said they, "the fecal matter run continually through a good cemented sewer with an abundant flow of water—the more water the better—and that solves the whole question." In answer to the inquiry, where shall the ingredients flow to, they replied, with an air of triumph, "to the nearest harbor or river pointed out by nature to receive our superfluous fluids." This all looked so very plausible that it was almost everywhere adopted. Enormous sums, particularly in England, were expended, not only for the building of sewers, but also for supplying the necessary amount of water for carrying it through the pipes, and the increase or decrease in the supply of water for this purpose was considered a measuring point of civilization.

But notwithstanding all well-meant laudations, it soon became apparent that the fundamental principle upon which the sewerage system of England was based was an entirely false one, and was attended with injurious effects even under the most favorable circumstances.

